# *In vivo* protective effects of chlorogenic acid against triptolide-induced hepatotoxicity and its mechanism

**DOI:** 10.1080/13880209.2018.1527370

**Published:** 2018-12-17

**Authors:** Jun-Ming Wang, Rong-Xing Chen, Lu-Lu Zhang, Ning-Ning Ding, Chen Liu, Ying Cui, Yong-Xian Cheng

**Affiliations:** a College of Pharmacy, Henan University of Chinese Medicine, Zhengzhou, China;; b Collaborative Innovation Center for Respiratory Disease Diagnosis and Treatment & Chinese Medicine Development of Henan Province, Henan University of Chinese Medicine, Zhengzhou, China;; c Health Science Center, Shenzhen University, Shenzhen, China

**Keywords:** Oxidative stress, antioxidant defence, Nrf2, hepatoprotection

## Abstract

**Context:** Triptolide (TP) has outstanding biological activities, but it induces toxicities, particular hepatotoxicity, severely limiting its clinical application. Chlorogenic acid (CGA) has prominently medicinal and nutritional values. However, until now, it is not known whether CGA could mitigate TP-induced hepatotoxicity.

**Objective:** This study explored the possible protection of CGA against TP-induced hepatotoxicity and its potential mechanisms, for the first time.

**Material and methods:** KM mice were treated orally with TP at a single dose of 1 mg/kg at 4 h after being treated with CGA (10, 20 and 40 mg/kg) for seven continuous days. Blood samples were collected at 24 h after TP administration for measurement of serum biomarkers, and hepatic tissues for analysis of potential mechanisms.

**Results:** TP treatment-induced acute hepatotoxicity manifested by the significant elevation in serum alanine transaminase (93.9 U/L), aspartate transaminase (185.8 U/L) and hepatic malondialdehyde (0.637 μmol/mg protein), and the remarkable reduction in hepatic glutathione (1.425 μg/mg protein), glutathione *S*-transferase, glutathione peroxidase, superoxide dismutase and catalase (91.7, 320.7, 360.6 and 140.7 U/mg protein, respectively). In contrast, pretreatment with CGA for 7 days effectively attenuated acute liver injury and oxidative stress caused by TP with each ED_50_ of 44.4, 57.1, 46.6, 22.2, 40.9, 58.1, 86.4 and 61.0 mg/kg, respectively. Furthermore, pretreatment with CGA promoted the accumulation of Nrf2 into the nucleus, and up-regulated mRNA expression of Nrf2-target downstream genes.

**Discussion and conclusions:** Combined CGA medication may probably reduce the risk of TP poisoning, and in-depth mechanisms can be developed around the signal molecules of Nrf2.

## Introduction

Triptolide (TP), a major active and toxic diterpenoid triepoxide isolated from the traditional Chinese herb, Radix *Tripterygium wilfordii* Hook f. (Celastraceae) (TWHF), and has exhibited multiple biological activities including anticancer, antirheumatoid arthritis, immunosuppressive, anti-inflammatory, antidiabetic nephropathy, antiproliferative and pro-apoptotic properties, etc. (Tao et al. 2012; Guo et al. 2016; Xiong et al. 2016; Ziaei and Halaby 2016; Zhang et al. 2017; Fan et al. 2018; Hu et al. 2018). However, it is well-known that due to the very narrow safety window of TP, it is easy to cause serious toxicity to digestive, reproductive, urinary and blood circulatory systems during use (Li et al. 2014; Xi et al. 2017). Among all organs, the liver is one of the most significant targets of TP-induced toxicities (Li et al. 2014; Xi et al. 2017).

Chlorogenic acid (CGA) is widely distributed in coffee, tea, medicinal plants and many fruits as an important natural dietary polyphenolic compound with multi-bioactivities such as hepatoprotection, antidiabetic nephropathy, antidiabetic retinopathy, antioxidant, antibacterial, cardioprotective, anti-inflammatory, antipyretic, neuroprotective, anti-obesity, antiviral, antimicrobial, antihypertension, free radicals scavenger and a central nervous system (CNS) stimulator (Xu et al. 2014; Feng et al. 2016; Ali et al. 2017; Bao et al. 2018; Mei et al. 2018; Naveed et al. 2018). However, it is unknown whether CGA could mitigate TP-induced hepatotoxicity and what is the underlying detoxication mechanism.

The current study observes the protective effects of CGA against TP-induced hepatotoxicity and its underlying mechanisms, for the first time.

## Materials and methods

### Experimental animals

Kunming (KM) male mice (18–22 g) were obtained from the Experimental Animal Center of Henan Province (Zhengzhou, China). Animals were given rodent laboratory chow and water *ad libitum* and maintained under controlled conditions with a temperature of 22 ± 1 °C, relative humidity of 60% ± 10%, and a 12 h light/dark cycle (lights on at 7:00 am.). All the procedures were in strict accordance with the P.R. China legislation on the use and care of laboratory animals and guidelines formulated by the Institute for Experimental Animals of Henan University of Chinese Medicine. The procedures were approved by the university committee for animal experiments.

### Reagents

TP was purchased from Nanjing Yuanzhi Biotechnology Co., Ltd. (Nanjing, China) with a purity of more than 98% as assayed by high-performance liquid chromatography (HPLC) analysis. CGA was purchased from Chengdu Mansite Biotechnology Co., Ltd. (Chengdu, China) with a purity of 99.39% as assayed by HPLC analysis. Kits including alanine/aspartate transaminase (ALT/AST), malondialdehyde (MDA), glutathione *S*-transferase (GST), glutathione peroxidase (GPx), superoxide dismutase (SOD) and catalase (CAT) were all purchased from Nanjing Jiancheng Bioengineering Institute (Nanjing, China).

### Treatment protocol

Mice were divided into the following 5 groups (*n* = 10). Control, TP only treated and TP + CGA treated (10, 20 and 40 mg/kg) (Wei et al. 2018). The control group received 0.5% sodium carboxymethylcellulose (CMC-Na) (0.2 mL per 10 g). Mice were given ig with CGA or 0.5% CMC-Na once daily for 7 days. Four hours after the last administration, except for the control group, mice in the other 4 groups were orally given a single dose of 1 mg/kg TP (Li et al. 2014; Wei et al. 2017). After 24 h of TP administration, the animals were harvested from the eyeballs and then sacrificed by decapitation. The liver tissues were quickly separated on the ice platform, and the large liver leaves were placed in 10% formalin fixative for pathological analysis. The remaining liver tissues were frozen at −80 °C to measure biological indicators.

### Assay for serum ALT and AST

Blood samples were obtained from mice of all groups (ten mice per group) for the determination of serum biochemical indicators. Serum ALT and AST were assayed by the commercial kits (Nanjing Jiancheng Bioengineering Institute, Nanjing, China) in accordance with the manufacturer’s protocols.

### Histological observation

After fixation in 10% formalin, the livers were examined for size, colour changes and haemorrhage. Slices of liver and kidney were cut into small pieces and histological sections were stained with hematoxylin and eosin (H&E) for the observation under the 100 times light microscopy.

### Assay for hepatic LPO level

Liver tissues were homogenized in cold physiological saline, respectively. Lipid peroxidation (LPO) was determined by the previously reported method (Högberg et al. 1974). Malondialdehyde (MDA) is an end product of LPO and serves as a means of quantifying LPO. MDA reacts with 2-thiobarbituric acid (TBA) to generate a pink-coloured product, which has an absorbance at 532 nm. LPO level was expressed as micromoles of MDA per milligram of protein based on tissue protein concentration through the Bradford assay (Nanjing Jiancheng Bioengineering Institute, Nanjing, China).

### Assay for hepatic GSH, GST, GPx, SOD and CAT levels

Liver tissues GSH, GST, GPx, SOD and CAT levels were determined by the commercial kits (Nanjing Jiancheng Bioengineering Institute, Nanjing, China) in accordance with the manufacturer’s protocols, and the results were all calculated based on tissue protein concentrations through the Bradford Protein Assay (Nanjing Jiancheng Bioengineering Institute, Nanjing, China).

### Western blot analysis

Proteins in hepatic tissues were separated as described in the Nuclear Extraction Reagent Kit (Pierce, USA). The protein concentrations were measured by BCA Protein Assay Kit (Beijing Solarbio Science & Technology Co., LTD., Beijing, China), and all the samples in the same experiment were normalized to the equal protein concentration. Protein samples were isolated by SDS-PAGE gel electrophoresis and transferred onto a PVDF membrane (Millipore, Billerica, MA, USA), and then incubated with the appropriate combination of primary and secondary antibodies, followed by ECL detection and quantification using an image analysis program. The grey densities of the protein bands were normalized by using Lamin B density as internal control, and the results were further normalized to control.

### Real-time PCR analysis

Total RNA was extracted from hepatic tissue using Trizol reagent following the manufacturer’s instructions. cDNA was synthesized and Real-time PCR was conducted as described in kits. Relative expression of target genes was normalized to glyceraldehydes-3-phosphate dehydrogenase (GAPDH), analyzed by 2^−ΔΔCt^ method and given as ratio compared with the control. The primer sequences are shown in Table 1.

**Table 1.** Primer sequences for Real-time PCR.

Target genes		Sequences	Accession No.	Size(bp)
GAPDH	Forward	5′-CAAGGTCATCCATGACAACTTTG-3′	NM_008084.3	90
	Reverse	5′-GGGCCATCCACAGTCTTCTG-3′		
HO-1	Forward	5′-ACGCATATACCCGCTACCTG-3′	NM_010442.2	174
	Reverse	5′-CCAGAGTGTTCATTCGAGCA-3′		
NQO1	Forward	5′-GCGTCTGGAGACTGTCTGGG-3′	M36660.1	170
	Reverse	5′-CGGCTGGAATGGACTTGC-3′		
GCLC	Forward	5′-AACACAGACCCAACCCAGAG-3′	NR_151778.1	201
	Reverse	5′-CCGCATCTTCTGGAAATGTT-3′		

### Statistical analysis

The results were presented as mean ± standard deviation of mean (SD). The differences among experimental groups were compared by one-way ANOVA (analysis of variance) followed by Least Significant Difference (LSD) (*p* < 0.05) using the SPSS (Statistics Package for Social Science) program Version 17.0.

## Results

### CGA mitigated TP-induced hepatotoxicity

**Figure 1.**
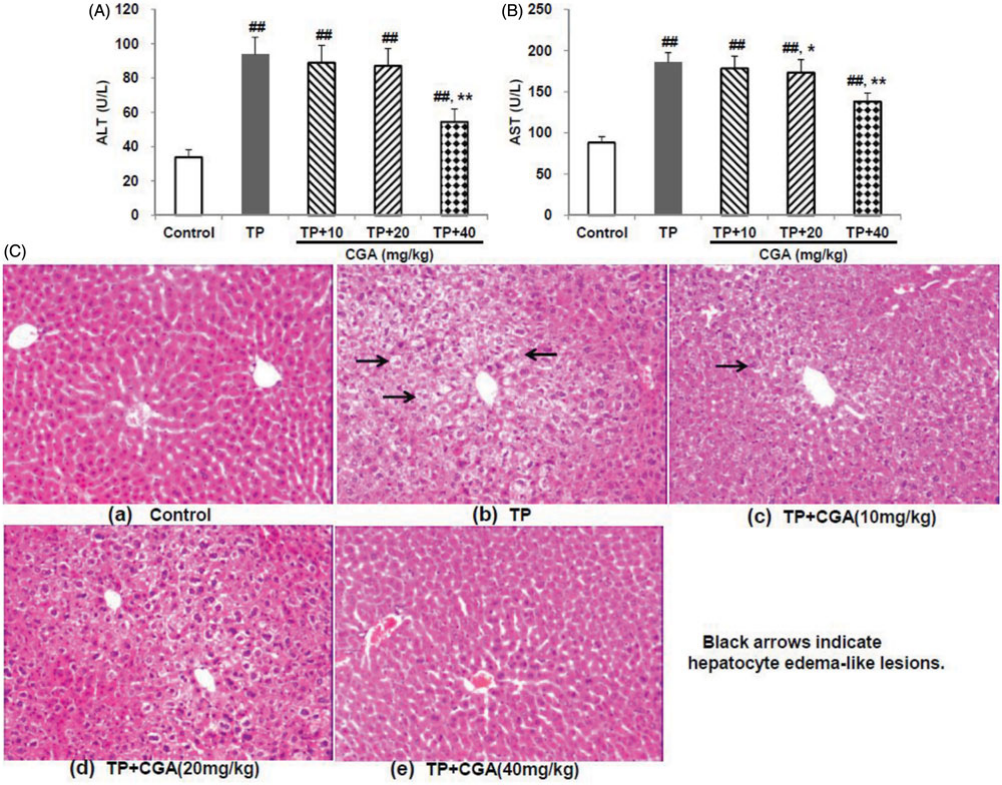
Effects of chlorogenic acid (CGA) on serum ALT (A) and AST (B) levels, and histopathology (C) for livers in triptolide (TP)-exposed mice. Data are presented as mean ± SD (*n* = 10). Significant differences compared with the control group were designated as ##*p* < 0.01, and with TP alone group as *p < 0.05 and **p < 0.01.

TP treatment-induced acute hepatotoxicity manifested by the significant elevation in serum ALT (93.9 U/L) (Figure 1(A)) and AST (185.8 U/L) (Figure 1(B)) levels (both *p* < 0.01). In contrast, pretreatment with CGA for 7 days effectively attenuated acute liver injury caused by TP with each effective half dose (ED_50_) of 44.4 and 57.1 mg/kg, respectively. Further, histological evaluation of the livers removed from mice treated with TP indicated hepatocyte oedema-like lesions (Figure 1(C)). After pretreatment with GCA at the doses of 20 and 40 mg/kg, these abnormal changes obviously decreased or even disappeared (Figure 1(C)). The results further corroborated the CGA inhibited TP-induced liver toxicity.

### CGA mitigated TP-induced abnormality of MDA, SOD and CAT

**Figure 2.**
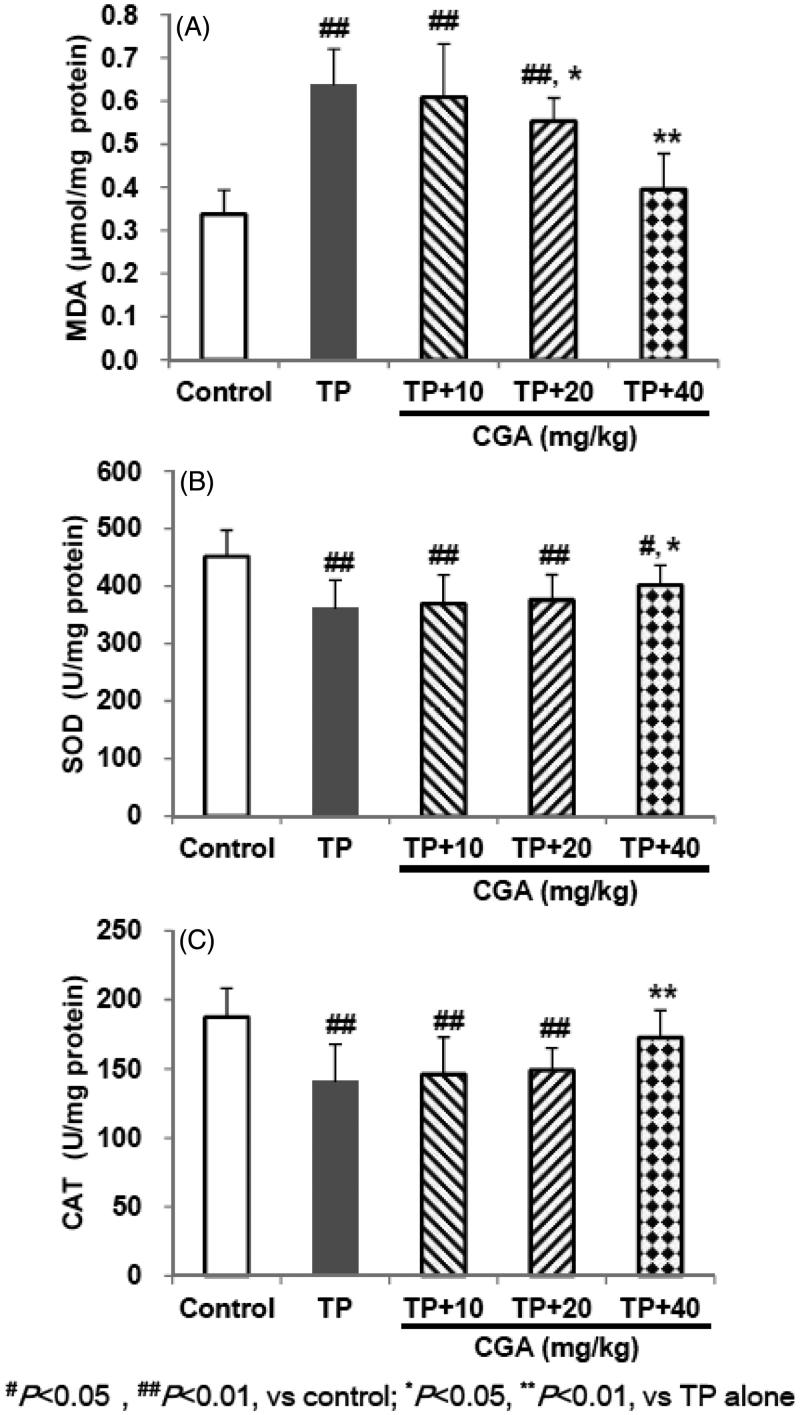
Effects of CGA on hepatic MDA (A), SOD (B) and CAT (C) levels in TP-exposed mice. Data are presented as mean ± SD (*n* = 10). Significant differences compared with the control group were designated as #*p* < 0.05 and ##*p* < 0.01, and with TP alone group as *p < 0.05 and **p < 0.01.

TP treatment caused lipid peroxidation and excessive depletion of major antioxidant enzymes manifested by the significant elevation in hepatic MDA (0.637 μmol/mg protein), and the remarkable reduction in hepatic SOD, and CAT levels (360.6 and 140.7 U/mg protein, respectively) (Figure 2(A–C)). In contrast, pretreatment with CGA for 7 days effectively attenuated hepatic lipid peroxidation and oxidative damage caused by TP with each effective half dose (ED_50_) of 46.6, 86.4 and 61.0 mg/kg, respectively.

### CGA prohibited TP-decreased GSH and it-related enzymes levels

**Figure 3.**
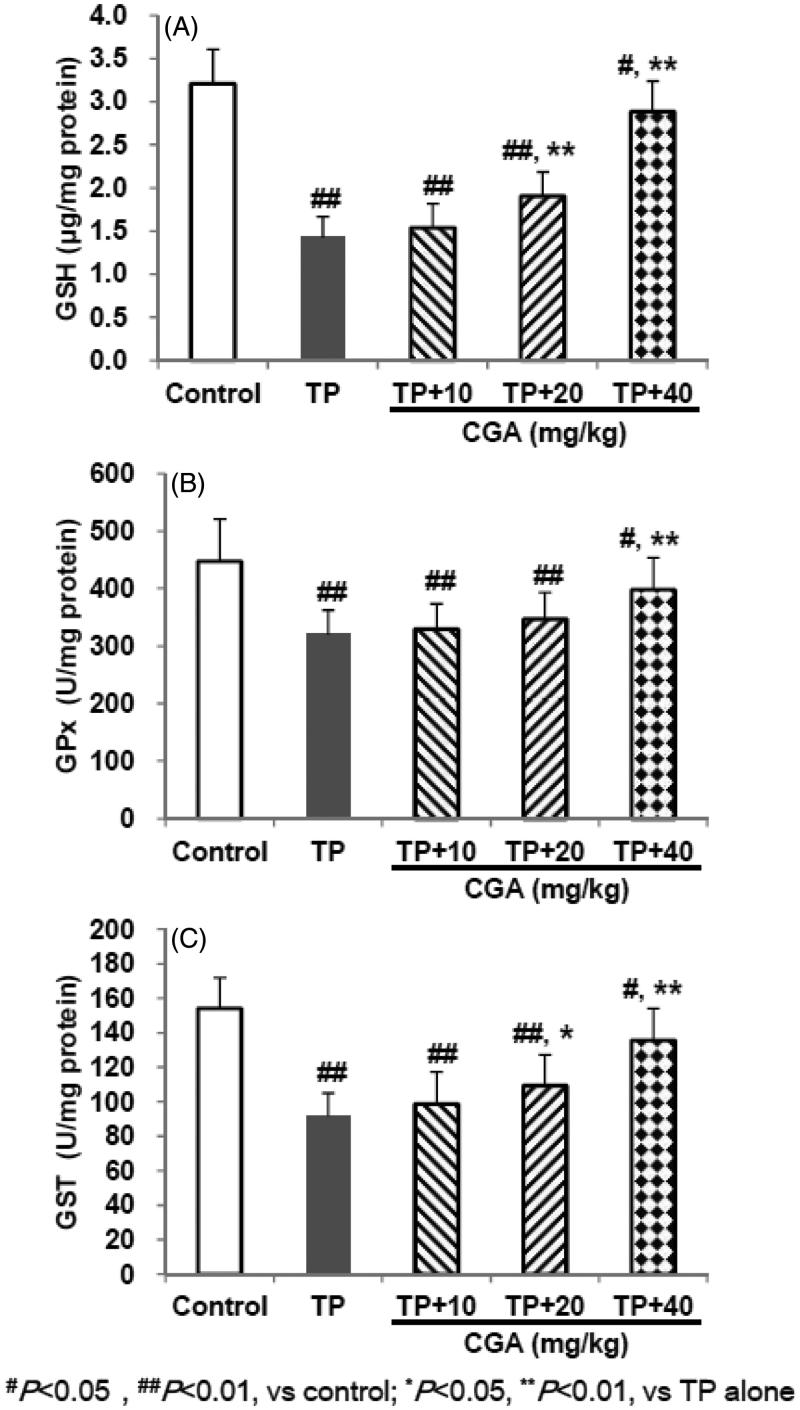
Effects of CGA on hepatic GSH (A), GPx (B) and GST (C) levels in TP-exposed mice. Data are presented as mean ± SD (*n* = 10). Significant differences compared with the control group were designated as #*p* < 0.05 and ##*p* < 0.01, and with TP alone group as *p < 0.05 and **p < 0.01.

TP treatment caused induced excessive depletion of GSH and its related enzymes manifested by the remarkable reduction in hepatic levels of GSH (1.425 μg/mg protein), GST and GPx (91.7 and 320.7 U/mg protein, respectively) (Figure 3(A–C)). In contrast, pretreatment with CGA for 7 days effectively attenuated hepatic GSH-related oxidative damage caused by TP with each effective half dose (ED_50_) of 22.2, 40.9 and 58.1 mg/kg, respectively.

### CGA activates the expression of Nrf2

**Figure 4.**
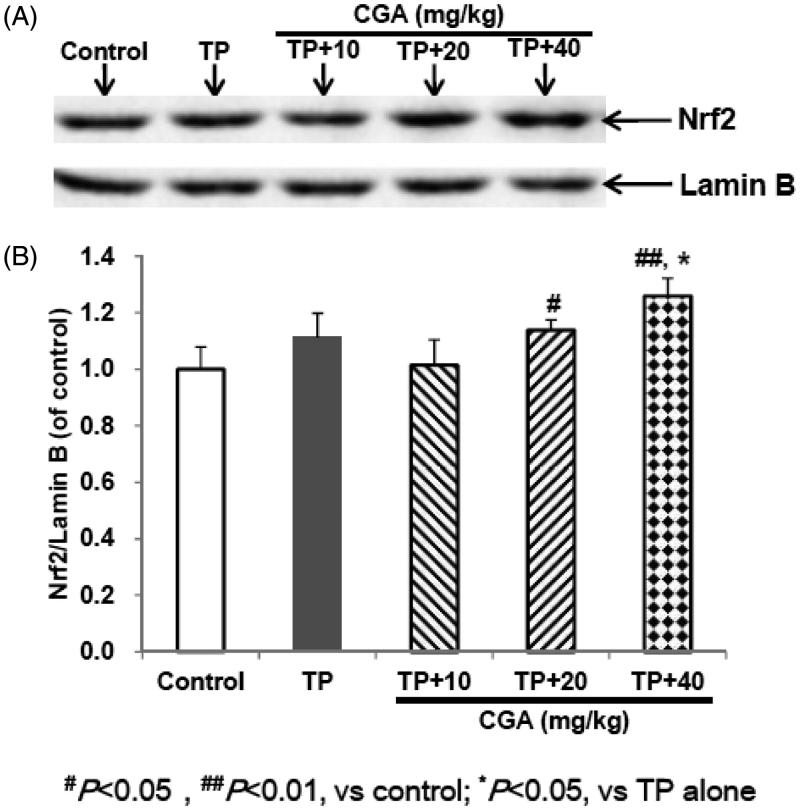
Effects of CGA on the protein expression of hepatic Nrf2 in TP-exposed mice. Data are presented as mean ± SD (*n* = 10). Significant differences compared with the control group were designated as #*p* < 0.05 and ##*p* < 0.01, and with TP alone group as *p < 0.05.

After administration of TP, the expression of Nrf2 protein in hepatic tissues of mice slightly increased compared with the control group, but there was no significant difference (Figure 4). After pretreatment with CGA at the dose of 40 mg/kg, whether compared with the control group or with TP alone group, Nrf2 protein expression was significantly up-regulated (*p* < 0.05). CGA treatment at the dose of 20 mg/kg promoted the accumulation of Nrf2 into the nucleus compared with the control group, but only slightly increased compared with TP alone group without significant difference. Whether compared with the control group or with TP alone group, after treatment with 10 mg/kg CGA, Nrf2 protein expression was slightly up-regulated, but no significant difference was observed. These results suggested that Nrf2 nuclear accumulation could involve the protection of CGA at the dose of 40 mg/kg against TP-induced liver toxicity.

### CGA up-regulates the expression of Nrf2-target downstream anti-oxidative genes

**Figure 5.**
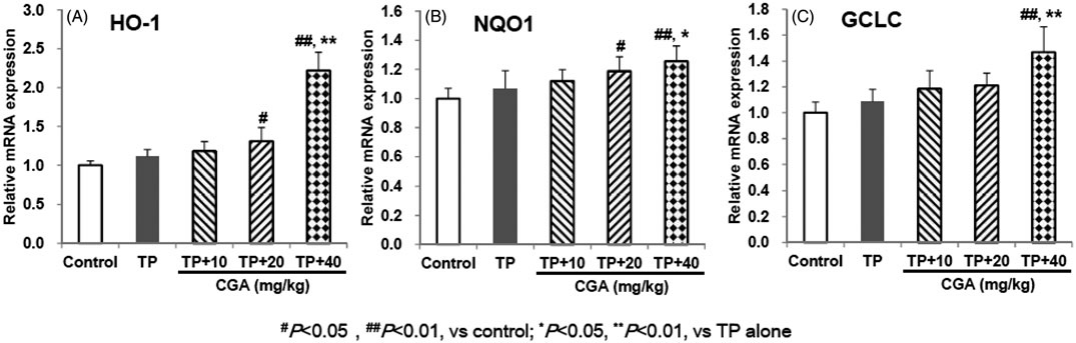
Effects of CGA on the mRNA expression of hepatic Nrf2-target downstream genes in TP-exposed mice. Data are presented as mean ± SD (*n* = 10). Significant differences compared with the control group were designated as #*p* < 0.05 and ##*p* < 0.01, and with TP alone group as *p < 0.05 and **p < 0.01.

After administration of TP, the expression of Nrf2-target genes including HO-1, NQO1 and GCLC all slightly increased compared with the control group, but there was no significant difference (Figure 5(A–C)). After pretreatment with CGA at the dose of 40 mg/kg, compared with TP alone group, the mRNA expression of HO-1, NQO1 and GCLC was significantly up-regulated (*p* < 0.01, *p* < 0.05 and *p* < 0.01, respectively). Besides, CGA treatment at the dose of 20 mg/kg significantly up-regulated the mRNA expression of HO-1 and NQO1 (both *p* < 0.05) instead of that of GCLC (*p* > 0.05) compared with the control group, but only slightly increased compared with TP alone group without significant difference (all *p* > 0.05). Whether compared with the control group or with TP alone group, after treatment with 10 mg/kg CGA, the mRNA expression of HO-1, NQO1 and GCLC was slightly up-regulated, but no significant difference was observed. These results suggested that Nrf2-target downstream anti-oxidative genes HO-1, NQO1 and GCLC could involve the protection of CGA at the dose of 40 mg/kg against TP-induced liver toxicity.

## Discussion

The significant elevation of serum ALT and AST, sensitive biomarkers for liver injury, commonly reflects hepatotoxicity (Pang et al. 2015; Xu et al. 2010). In the present study, TP-induced significant elevation of serum ALT and AST, while after pretreatment with CGA their elevation was obviously reversed in a dose-dependent manner. The result indicated that TP-caused hepatotoxicity was alleviated by CGA, which was further evidenced by the histopathological analysis results.

The common pathophysiology of liver disease is oxidative stress induced by reactive oxygen species (ROS) (Pang et al. 2015; Shi et al. 2016). Cellular oxidative stress often happens during imbalance between oxidants and antioxidants, furthermore, the levels of many antioxidant-related enzymes and non-enzymatic antioxidants may be changed during the pathogenesis of liver toxicity (Pang et al. 2015; Shi et al. 2016; Roh et al. 2018). Among them, LPO is a free radical-mediated process (Shi et al. 2016). MDA, as one of the main end products of LPO, has the characterization of cross-linking cellular macromolecules including protein or DNA and causes widespread cellular damage (Pang et al. 2015; Shi et al. 2016), and its marked increase usually indicates LPO damage. SOD and CAT participate in defending oxidative stress injury as intracellular main antioxidant enzymes (Shi et al. 2016). GSH helps protect cells against ROS such as free radicals and peroxides as an antioxidant (Pang et al. 2015; Shi et al. 2016). Its excessive exhaustion can induce oxidative stress injury. GST and GPx, as intracellular GSH-related enzymes, cooperate with GSH in participating during the oxidative stress injury as well (Roh et al. 2018). Previous studies reported that CGA could protect liver toxicities induced by acetaminophen or carbon tetrachloride (CCl_4_) via the inhibition of oxidative stress evidenced by decreased MDA level and (or) increased GSH, SOD and CAT levels in liver tissues (Pang et al. 2015; Shi et al. 2016). Another study reported that CGA attenuated isoproterenol-induced myocardial oxidative stress in rat myocardium, evidenced by increased GSH, GST, GPx, SOD and CAT levels in the erythrocytes, plasma and heart tissue of rats (Akila and Vennila 2016). In the current study, TP administration induced significant elevation of MDA, and remarkable reduction of SOD, CAT, GSH, GPx and GST levels, while after pretreatment with CGA, the abnormality of these six indicators were all significantly reversed in dose-dependent manners to some extent, which suggested that CGA could alleviate TP-induced liver toxicity probably by inhibiting LPO damage, enhancing endogenous antioxidant enzymes, and thereby resisting oxidative stress.

Nrf2 regulates encoding antioxidant proteins by interacting with Antioxidant Response Element (ARE) and is the most important endogenous anti-oxidative stress pathway that is currently found (Li et al. 2014; Wei et al. 2017). Physiologically, Nrf2 binds to its inhibitory protein Kelch-like ECH associated protein 1 (Keap1) in the cytoplasm, failing to enter the nucleus to exert its transcriptional activity (Li et al. 2014; Wei et al. 2017). Oxidative and electrophilic stresses cause dissociation of Nrf2 from Keap1 and lead Nrf2 to translocate into the nucleus where it can bind to ARE, activate the expression of downstream Nrf2-target genes such as HO-1, NQO1 and GCLC, and thereby play the role of antioxidant damage (Li et al. 2014; Wei et al. 2017). In the present study, pretreatment with CGA promoted the accumulation of Nrf2 into the nucleus, and up-regulated the mRNA expression of Nrf2-target genes HO-1, NQO1 and GCLC, indicating that the activation of Nrf2 and up-regulation of Nrf2-target downstream genes expression could involve the protection of CGA against TP-induced liver toxicity.

Collectively, the present study demonstrates that CGA mitigates liver toxicity induced by TP, and the potential mechanisms could probably involve the activation of Nrf2, the up-regulated expression of Nrf2-target downstream genes such as HO-1, NQO1 and GCLC, the enhancement endogenous antioxidant enzymes, the inhibition of LPO damage, and thereby the improvement of body’s antioxidant defence systems and the resistance to oxidative stress.
